# Artificial Intelligence–Derived 3D Body Composition Analysis of the Entire Lumbar Region From CT Scans Reveals Variation Across Disease Stages at Colorectal Cancer Diagnosis

**DOI:** 10.1155/rrp/6057943

**Published:** 2026-08-30

**Authors:** Ke Cao, Lara Wirth, Susan Teo, Josephine Yeung, Paul N. Baird, Justin M. C. Yeung

**Affiliations:** ^1^ Department of Surgery, Western Precinct, University of Melbourne, Melbourne, Australia, unimelb.edu.au; ^2^ Department of Surgery, University of Melbourne, Melbourne, Australia, unimelb.edu.au; ^3^ Department of Colorectal Surgery, Western Health, Melbourne, Australia, westernhealth.org.au

**Keywords:** artificial intelligence, body composition, colorectal cancer, computed tomography

## Abstract

**Purpose:**

While changes in weight are often observed in patients with colorectal cancer (CRC), they provide limited information regarding how or if body composition changes might occur. We used artificial intelligence (AI) to analyse computed tomography (CT) images from patients’ lumbar region to investigate how body composition differs across clinical disease stages of CRC.

**Methods:**

A retrospective analysis was performed on patients diagnosed with CRC and treated at Western Health, Melbourne (2013–2023). Three‐dimensional measures of skeletal muscle (SM), visceral adipose tissue (VAT), subcutaneous adipose tissue (SAT) volumes and radiodensities were automatically segmented from staging CT scans (L1–L5) using a pretrained AI model. Disease stage was determined using the Australian Clinicopathological Staging (ACPS) system. Body composition values were adjusted for physiological variation (age, sex and height) and compared across ACPS stages.

**Results:**

A total of 1138 individuals were evaluated (68.2 ± 13.0 years; 59.8% male), with 24% classified as Stage A, 30.5% as Stage B, 17.5% as Stage C, and 28% as Stage D. No significant differences were observed between Stages B and C, and these groups were therefore combined (Stage B/C) for further analysis. VAT volume was significantly lower in Stage B/C versus Stage A (*p* = 0.004) and declined further in Stage D (*p* = 0.003). In contrast, SM and SAT volumes were significantly lower only in Stage D compared with earlier stages. Radiodensity measures showed that SAT and VAT radiodensity increased progressively from Stage A to Stage D, whereas SM radiodensity increased only in Stage D.

**Conclusion:**

CRC patients were shown to exhibit distinct body composition changes at diagnosis depending on disease stage. Adipose tissue loss and density changes occurred earlier than muscle changes. Longitudinal studies are warranted to understand these trajectories but suggest that nutritional and supportive interventions could be targeted based on CRC stage at diagnosis.

## 1. Introduction

Colorectal cancer (CRC) is the third most prevalent cancer worldwide and is the second leading cause of cancer‐related mortality [1]. In recent years, body composition analysis has been increasingly conducted, using computed tomography (CT) scans that are routinely acquired as part of the standard clinical care. These cross‐sectional images offer a valuable opportunity to assess body composition, particularly the quantity and distribution of abdominal lean (e.g., muscle) and adipose tissues in CRC patients [2, 3].

As a direct measure of the body’s protein and energy reservoir, body composition is a key determinant of metabolic health and functional capacity, and its assessment is essential to guide and monitor nutritional and metabolic support [4–6]. This is important in CRC therapy, as both the disease itself and the prolonged treatment process, including surgical resection, chemotherapy, radiotherapy, or molecular targeted therapy, can lead to a range of symptoms. These include appetite loss, anaemia, impaired immunity, and unexpected weight loss, resulting in metabolic changes and malnutrition [7, 8]. These conditions are associated with poorer treatment response and worse clinical outcomes [9]. Once irreversible cachexia develops, expectancy in CRC patients can become limited. There is strong evidence to support prehabilitation, where metabolic and nutritional status of CRC patients is assessed and optimised before any therapeutic intervention is undertaken [9].

To enable early detection of nutritional disturbances, the European Society for Clinical Nutrition and Metabolism (ESPEN) recommends regular evaluation of nutritional intake, weight change, and body mass index (BMI) at diagnosis [5, 10]. However, ESPEN also cautions that a cancer diagnosis alone should not be considered sufficient indication for initiating nutritional supplementation. This contradiction underscores the inadequacy of current tools such as weight and BMI tracking, which, while common in practice, cannot reveal specific changes in body composition [11]. A previous study by Brown et al. [12] showed that weight stability masked clinically meaningful skeletal muscle (SM) depletion in CRC. Thus, a gap remains in understanding how weight relates to body composition changes present at diagnosis and limits the development of earlier and more targeted interventions.

Advances in artificial intelligence (AI) have enabled fully automated extraction of body composition measurements from routine CT scans, thereby greatly streamlining assessment of these tissues. These developments extend body composition analysis beyond the traditional single‐slice CT assessment at the third lumbar vertebra, allowing evaluation across multiple slices or the entire lumbar region to provide a more comprehensive three‐dimensional (3D) profile. Our recent studies demonstrated the added value of assessing the complete lumbar region compared with only a single CT slice in CRC [13]. Additionally, body composition measures are also strongly influenced by demographic factors such as age, sex, and body size [14], and these should also be taken into account when investigating how nutritional or other targeted interventions may be administered.

## 2. Methods

This retrospective study was conducted at a single tertiary referral centre, Western Health, Melbourne, Australia. The study was approved by the Western Health Office for Research (project QA2020.24_63907). The protocol followed the tenets of the Declaration of Helsinki, and all privacy requirements were met.

### 2.1. Subjects

Data were collected from participants diagnosed with CRC at Western Health, Melbourne, Australia, between 2013 and 2023. Eligible patients were identified from the Australian Comprehensive Cancer Outcomes and Research Database (ACCORD), a prospective clinical registry for CRC patients within Victoria, Australia.

Patients were included if they met the following criteria: (a) a confirmed diagnosis of CRC at Western Health during the study period and (b) availability of axial staging CT scans at hospital admission and performed prior to any treatment.

Patients with suboptimal CT image quality that prevented accurate segmentation were excluded from the study. Patients with no available clinical or demographic information were also excluded.

### 2.2. CT Collection and Body Composition Assessment

The CT acquisition protocols and preprocessing steps for body composition assessment have been detailed in a previous study [13]. A routinely collected CT scan was used for each patient at initial hospital admission and before treatment regimens were administered. CT scans were acquired mainly using GE HealthCare scanners, with X‐ray tube voltages ranging from 80 to 140 kVp. The CT parameters varied across examinations, reflecting routine clinical practice in which scanning settings were selected according to the clinical requirements. Disease diagnosis and staging were made following clinical, histological and radiological assessment.

A pretrained and validated AI‐based segmentation model was used to automatically identify and quantify SM, visceral adipose tissue (VAT) and subcutaneous adipose tissue (SAT). The development and validation of the segmentation model have been previously described [15].

Body composition was assessed using all CT slices covering the full lumbar region of the spine (L1–L5, including intervertebral regions). This allowed for the quantification of SM, VAT and SAT volumes (cm^3^), as well as their respective radiodensities (Hounsfield units). The procedures for calculating tissue volume and radiodensity have been detailed in our prior studies [13, 16].

### 2.3. Demographic and Clinical Data Collection

Demographic data, including age (years), sex, height (cm), weight (kg) and BMI were obtained from the ACCORD registry. Cancer stage was classified according to the Australian Clinicopathological Staging (ACPS) system and was also retrieved from the ACCORD registry. Table 1 outlines the definitions of each ACPS stage alongside their TNM equivalents [17].

**TABLE 1:** ACPS staging and corresponding TNM classification for CRC [17].

ACPS stage	Clinical description	TNM equivalent
Stage A	The tumour has invaded several layers of the bowel wall but has not extended beyond it	T1 or T2, N0 M0
Stage B	The cancer has grown through the muscle layer of the bowel or rectum and into nearby tissues, without spreading to the lymph nodes	T2 or T4, N0 M0
Stage C	The cancer has spread to nearby lymph nodes but has not metastasised to distant parts of the body	Any T, N1 or N2, M0
Stage D	Known as metastatic bowel cancer. The cancer has spread from where it started in the colon or rectum to other organs	Any T, any N, M1

### 2.4. Analysis

Data were analysed with RStudio (Version 2022.2.2.485) for Windows. All statistical tests were considered significant when the *p* value was less than 0.05. One‐way analysis of variance (ANOVA) was used to assess differences in age, height, weight, BMI and individual body composition measures across ACPS stages, while the chi‐squared (*χ* ^2^) test was applied to evaluate differences in sex distribution across stages. Where overall ANOVA results were significant, post hoc comparisons were conducted using Tukey’s honest significant difference (TukeyHSD) test to identify specific between‐group differences. Homogeneity of variance was assessed using Levene’s test for body composition measures, body weight and BMI.

All body composition measurements were adjusted for age, sex and height prior to comparisons across cancer stages. This adjustment aimed to isolate disease‐related variation by mitigating physiological influences. For each body composition parameter (Y), a separate linear regression model was fitted with age, sex and height as predictors. The predicted value for each participant was used to estimate the expected physiological measurement, and residuals were calculated as the difference between the observed and predicted values (Residual = Y_observed − Y_predicted). These residuals represented adjusted body composition values capturing deviations from what would be expected based on each participant’s age, sex and height.

Only participants with complete data for all required clinical and demographic variables were included in the comparison. The resulting residuals were standardised to facilitate direct comparisons across ACPS stages.

## 3. Results

A total of 1138 CRC patients were included in the analysis. About 40.2% of the participants were female, and the mean age was 68.2 (standard deviation; SD = 13.0) years.

Staging data were available for 970 subjects, of which 234 (24%) were classified as Stage A, 296 (30.5%) as Stage B, 170 (17.5%) as Stage C and 270 (28%) as Stage D. The mean age (Table 2 and Supporting Figure 1) differed significantly across stages (*p* < 0.001), with Stage D patients being the youngest (4.5 years younger than Stage A), averaging 63.25 ± 14.27 years. No significant difference was observed in sex distribution across the four stages (*p* = 0.06). Height data were available for 867 patients, and patients with Stage D disease were significantly taller (3.5 cm) than those with Stage C disease (*p* = 0.008). Demographics for each stage are shown (Table 2).

**TABLE 2:** Demographics for each stage.

	Stage A (*n* = 234)	Stage B (*n* = 296)	Stage C (*n* = 170)	Stage D (*n* = 270)	*p*
Age	67.86 ± 10.91	69.25 ± 12.38	70.91 ± 11.88	63.25 ± 14.27	**< 0.001** A–D: < 0.001 B–D: < 0.001 C‐D: < 0.001

Sex (female, *n* (%))	85 (36.3%)	126 (42.6%)	81 (47.6%)	99 (36.7%)	0.06

Height (cm)	167.0 ± 10.07	166.28 ± 9.63	164.77 ± 10.55	168.13 ± 9.74	**0.01** C‐D: 0.008

*Note:* For each variable, the *p* value in the first row represented the overall comparison across all stages. If this comparison was statistically significant, pairwise comparisons were conducted, and only those with significant differences were included in the table. Bold values in the first row for each variable indicate statistical significance.

**FIGURE 1:**
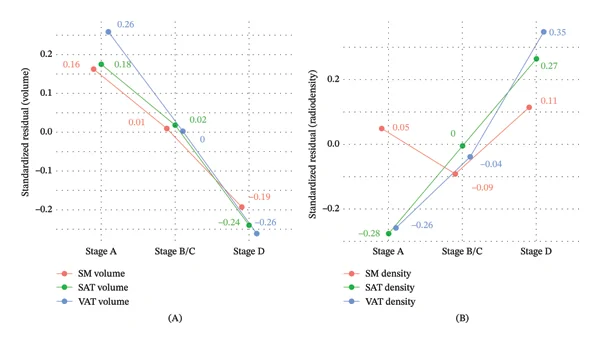
Mean standardised residuals of SM, SAT and VAT volume (A) and radiodensity (B) across ACPS stage, with Stages B and C combined.

Body composition measures, adjusted for age, sex and height using multivariable regression residuals (Supporting Table 1 presented the coefficients from the regression models for each body composition parameter) demonstrated stage‐specific differences at the time of diagnosis (Supporting Table 2). Supporting Table 2 showed no significant differences in any body composition measures between Stage B and Stage C after adjustment, we therefore grouped these two stages together and repeated the analysis (Table 3 and Figure 1).

**TABLE 3:** Weight, BMI and residualised body composition measures by ACPS stage, with Stages B and C combined.

	Stage A (*n* = 234)	Stage B/C (*n* = 466)	Stage D (*n* = 270)	*p* values		
				A vs. B/C	A vs. D	B/C vs. D
Weight (kg)	1.02 ± 0.23	0.95 ± 0.23	0.96 ± 0.26	↓↓	↓	—
BMI	1.02 ± 0.19	0.97 ± 0.21	0.94 ± 0.23	↓	↓↓↓	—
SM volume residual	0.16 ± 0.95	0.01 ± 0.99	−0.19 ± 1.04	—	↓↓↓	↓
SM density residual	0.05 ± 1.02	−0.09 ± 1	0.11 ± 0.97	—	—	↑
SAT volume residual	0.18 ± 0.88	0.02 ± 0.99	−0.24 ± 1.09	—	↓↓↓	↓↓
SAT density residual	−0.28 ± 0.72	0 ± 0.98	0.27 ± 1.19	↑↑	↑↑↑	↑↑
VAT volume residual	0.26 ± 1.06	0 ± 0.94	−0.26 ± 0.98	↓↓	↓↓↓	↓↓
VAT density residual	−0.26 ± 0.87	−0.04 ± 0.99	0.35 ± 1.04	↑	↑↑↑	↑↑↑

*Note:* Weight and BMI were standardised to allow comparison across ACPS stages. Residuals were obtained from linear regression models adjusted for age, sex and height. Values presented as mean ± SD of the standardised values for each stage. Post hoc pairwise comparisons were symbolised as ↑↑↑/↓↓↓ for *p* < 0.001, ↑↑/↓↓ for *p* < 0.01, ↑/↓ for *p* < 0.05; — for not significant (*p* ≥ 0.05). Arrows indicated the direction of the difference.

### 3.1. Variation in Muscle Across Stages

SM volume was highest in Stage A (0.16 ± 0.95), intermediate in Stage B/C (0.01 ± 0.99), and lowest in Stage D (−0.19 ± 1.04). Significant declines were observed between Stage A and Stage D (*p* < 0.001) and between Stage B/C and Stage D (*p* = 0.04) but not between Stage A and Stage B/C (*p* = 0.14).

SM radiodensity declined from Stage A (0.05 ± 1.02) to Stage B/C (−0.09 ± 1.00), although this difference was not significant (*p* = 0.21). Of interest was an observed significant increase between Stage B/C (−0.09 ± 1.00) and Stage D (0.11 ± 0.97) (*p* = 0.04).

### 3.2. Variation in SAT Across Stages

Similar to SM volume, SAT volume was highest in Stage A (0.18 ± 0.88), intermediate in Stage B/C (0.02 ± 0.99) and lowest in Stage D (−0.24 ± 1.09). Significant declines were observed between Stage A and Stage D (*p* < 0.001) and between Stage B/C and Stage D (*p* = 0.004). No significant difference was found between Stage A and Stage B/C (*p* = 0.13).

SAT radiodensity showed a significant increase from each stage to the next, rising from Stage A (−0.28 ± 0.72) to Stage B/C (0.00 ± 0.98, *p* = 0.002), and further to Stage D (0.27 ± 1.19, *p* = 0.003 for B/C‐D and *p* < 0.001 for Stages A–D).

### 3.3. Variation in VAT Across Stages

VAT volume showed a significant decrease from each stage to the next, reducing from Stage A (0.26 ± 1.06) to Stage B/C (0.00 ± 0.94, *p* = 0.004), and further to Stage D (−0.26 ± 0.98, *p* = 0.003 for B/C‐D and *p* < 0.001 for Stages A–D).

VAT radiodensity showed a significant increase from each stage to the next, rising from Stage A (−0.26 ± 0.87) to Stage B/C (−0.04 ± 0.99, *p* = 0.02), and further to Stage D (0.35 ± 1.04, *p* < 0.001 for B/C‐D and *p* < 0.001 for Stages A–D).

### 3.4. Variation in Weight and BMI Across Stages

Significant decreases in both weight and BMI were observed between Stage A and Stage B/C, as well as between Stage A and Stage D. No significant differences were found between Stage B/C and Stage D (*p* = 0.99 for weight and *p* = 0.12 for BMI).

## 4. Discussion

In this study, we compared six body composition measurements, including both muscle and adipose tissues (VAT and SAT), across clinical stages of CRC using patients’ initial CT scans at hospital presentation. Our results revealed stage‐specific trajectories of body composition change already evident at diagnosis, characterised by reductions in SM, VAT and SAT volumes from early to late stage, together with a continual rise in adipose tissue radiodensity and nonlinear changes in muscle radiodensity, suggesting underlying tissue remodelling, inflammation or fibrosis. Adipose tissue changes appeared earlier, with significant reductions in VAT volume and increases in VAT and SAT radiodensity observed as early as Stage B/C. In contrast, significant muscle loss was only evident at Stage D, when metastases were typically present. Importantly, these changes occurred even in the absence of significant reductions in weight and BMI, which are commonly used in clinical practice to evaluate cancer‐related depletion. Although weight and BMI decreased significantly from Stage A to later stages, they failed to discriminate between Stage B/C and Stage D (*p* = 0.99 for weight; *p* = 0.12 for BMI), where significant loss in tissue volume and increase in radiodensity were observed across muscle, VAT and SAT (Table 3).

CRC is a global health challenge, accounting for 2 million new cases and approximately 1 million deaths annually [18]. Once considered primarily a disease of older adults, it is now increasingly prevalent among younger generations. In early 2023, the American Cancer Society (ACS) reported that around 20% of CRC diagnoses in 2019 occurred in patients under the age of 55, which was nearly double the rate reported in 1995 [19]. Our data aligned with these findings, with more than 18% of patients diagnosed before the age of 55. Alarmingly, our data also suggested that 45% of these younger patients presented to hospital with advanced‐stage disease (ACPS stage D), indicating the presence of metastases at diagnosis. Although the exact reasons remain unclear, several possible risk factors, including sedentary lifestyle, increased consumption of processed foods and red meat, may contribute to this concerning rise [20]. This shift toward younger onset and more advanced disease presentation underscores the critical importance of screening, early detection and timely intervention to improve patient survival outcomes.

Metabolic changes, often resulting in nutritional issues, are common among patients with CRC, and these adversely influence clinical outcomes [9]. Tumour growth is an energy‐intensive process that worsens nutritional status through appetite loss, immune dysfunction and the release of inflammatory molecules that disrupt hunger and satiety regulation [9, 21]. These disturbances are further influenced by CRC treatments, including chemotherapy, radiotherapy and surgical resection, which may impair bowel absorption and exacerbate metabolic decline through multiple mechanisms. Collectively, these challenges compromise patients’ health and drive body changes, including muscle and fat, that mediate through cancer‐related alterations in lipid and protein metabolism. Such changes may contribute to malnutrition, anorexia, cachexia and sarcopenia, all of which negatively affect clinical outcomes [8]. Therefore, early metabolic and nutritional assessment before therapeutic intervention, together with continuous monitoring throughout the disease trajectory is important.

Body composition measures are essential tool to effectively evaluate nutritional status [11]. Understanding these changes at the time of diagnosis, the earliest point at which clinical intervention can be initiated is therefore critical. Our results indicated that early nutritional support should be directed toward adipose tissues. This was consistent with previous observations showing that fat depletion in cancer patients was often more pronounced than, and could precede, muscle loss [22, 23]. VAT appeared to represent the earliest site of depletion, with significant reductions observed as early as Stage B/C. This might have been explained by the high metabolic activity of VAT [24] which exhibited elevated lipolytic activity due to a greater density of β1‐, β2‐ and β3‐adrenergic receptors [25]. These features likely rendered VAT particularly vulnerable to early breakdown. Along with VAT loss, both VAT and SAT radiodensity increased significantly at Stage B/C and continued to rise with advancing stage, a pattern previously linked to a poor prognosis in cancer patients [26]. This likely reflects tissue remodelling processes such as inflammation [27], fibrosis or reduced lipid content within adipocytes [26]. These findings suggest that anti‐inflammatory nutritional strategies or other targeted interventions aimed at controlling systemic inflammation and increasing energy intake should be considered in patients diagnosed at these stages to help prevent further adipose tissue depletion.

Although muscle loss has been widely recognised in CRC and is considered a risk factor for mortality [28, 29], most previous studies have assessed changes longitudinally after the diagnosis, when muscle depletion may be influenced by treatments or by medical or surgical complications. Our results suggest the presence of muscle loss across all stages at diagnosis, but a significant reduction was only observed at the advanced stage (Stage D), when metastases were present. These findings support the concept that, once metastatic spread has occurred, clinical care should also include strategies to preserve or improve muscle in addition to addressing adipose tissue depletion. Interestingly, muscle density showed a significant increase at Stage D. This contrasted with the conventional understanding where lower muscle radiodensity, which may result from myosteatosis or fat infiltration, is typically associated with the worse prognosis [30]. Based on this, muscle density would be expected to be lowest in advanced disease. Our findings partially align with this expectation, showing a decline prior to the metastatic stages; however, a significant increase emerged at Stage D (the metastatic stage). This rise may reflect a complex interplay of factors driven by disease burden, potentially involving dehydration and reduced intramuscular water content [31] or potential selective depletion of fat‐infiltrated or necrotic fibres during severe muscle wasting. These mechanisms warrant further investigation to clarify their relative contributions to muscle quality changes across disease progression.

Recent weight loss, current BMI, food intake history and nutritional assessment questionnaires are commonly used by clinicians and dietitians during routine assessments [11]. While these measures are simple and practical, they are subject to recall bias, lack sensitivity for detecting subtle changes in nutritional status and cannot differentiate between specific body tissues. Our data also showed a loss in weight and BMI form Stage A to later stages; however, it shows no significant difference between later stages (Stage B/C and Stage D). During this period, however, significant changes occurred in the volume and radiodensity of muscle, as well as in both VAT and SAT. These changes did not translate into differences in body weight, which might be explained by factors such as fat redistribution, tumour growth or changes in other parts of the body. These results further demonstrate that weight alone is an insensitive marker of disease‐related body composition changes.

A residual approach was applied in this study to account for the influence of demographic variation on body composition. This adjustment was essential, as each body composition parameter is differentially associated with age, sex and height as a proxy for body size. Reliance on raw values alone would make it difficult to distinguish whether observed differences were attributable to demographic variation or disease effects. Weight‐related measures (e.g., weight, BMI, BSA) were intentionally excluded as adjustment variables, as these may themselves be outcomes of the disease process rather than true demographic confounders. We acknowledged that not all potential confounders were excluded, as body composition could be influenced by multiple factors; however, our approach mitigated the impact of the most prominent demographic variables and enhanced the validity of the disease‐specific associations observed. In the meantime, the residual adjustment model assumed linear relationships between patient characteristics and body composition and did not fully capture potential nonlinear relationships or interaction effects among these factors. Future studies are expected to investigate more flexible modelling approaches, such as nonlinear or interaction models, to improve adjustment and enhance the interpretation of body composition measures in patients with CRC. This further highlighted the need to establish reference standards for body composition at different stages of CRC diagnosis. At present, no consensus exists on what constitutes normal or expected body composition values for nutritional assessment in this context. Such standards would allow clinicians to determine the extent of deviation for each patient, quantify tissue loss and guide the delivery of personalised care.

### 4.1. Limitations

There were several limitations to this study. This is a cross‐sectional rather than prospective study. While following changes in body composition in a patient would be valuable, treatment regimens would impact on any subsequent measures. Thus, we chose to look at CT scans at disease presentation and obtain group data at each stage. Thus, this study cannot comment on individual changes over time. Data for this study were collected at a single centre. Although conducting the study at a single centre helped maintain consistency in patient identification and data collection procedures, the findings may be less generalisable to other healthcare settings with different patient populations or clinical workflows. To enhance the clinical relevance of our findings, we retained as many eligible participants as possible without restricting recruitment to specific clinical settings, thereby allowing the study cohort to better reflect routine clinical practice. Nevertheless, external validation using independent cohorts from other institutions is warranted to confirm the generalisability and robustness of our findings. Furthermore, although the primary aim was to characterise body composition changes across CRC stages, the inclusion of age‐ and sex‐matched healthy controls in future studies would provide valuable reference data and further improve our understanding of body composition changes, particularly their trajectories and relationships with patient characteristics in CRC. In addition, although this study included CRC patients across all stages, significant body composition changes may occur prior to first presentation. We also cannot indicate how these findings compare to individuals without CRC.

## 5. Conclusion

The body composition of CRC patients differs by stage at diagnosis. Patients at earlier stages tended to have decreased VAT volume and increased adipose tissue density, whereas those with advanced disease demonstrated significantly lower muscle volume. These body composition differences were significant even though body weight and BMI changes were not significant indicating that body composition changes are not being adequately accounted for. Prehabilitation targeting these stage‐specific changes should be developed and rigorously evaluated for their potential to improve clinical outcomes and, ultimately, patient survival and quality of life.

## Funding

No specific funding was acquired for this study. Open access publishing facilitated by The University of Melbourne, as part of the Wiley ‐ The University of Melbourne agreement via the Council of Australasian University Librarians.

## Disclosure

The sponsor or funding organisations had no role in the design or conduct of this research.

## Conflicts of Interest

The authors declare no conflicts of interest.

## Supporting Information

Additional supporting information can be found online in the Supporting Information section.

## Supporting information


**Supporting Information 1** Supporting Figure 1: Distribution of patient age across stages. Each violin represents the age distribution for a given stage (A–D). The outer shape of the violin shows the probability density of the data at different ages. The box inside the violin represents the interquartile range (IQR), with the thick horizontal line marking the median age. The white diamond indicates the mean age for each stage.

**Supporting Information 2** Supporting Table 1: Coefficients from the regression models for each body composition parameter. Supporting Table 2: Residualised body composition measures by ACPS stage. Residuals were obtained from linear regression models adjusted for age, sex and height. Values presented as mean ± SD of the standardised values for each stage. The *p* value of each variable indicates the overall difference across all stages. Where this overall comparison was statistically significant, pairwise comparisons were performed, and only significant pairwise results are reported in the table.

## Data Availability

The data that support the findings of this study are available from the corresponding author upon reasonable request.
