# Supplementary material for: Artificial Intelligence–Derived 3D Body Composition Analysis of the Entire Lumbar Region From CT Scans Reveals Variation Across Disease Stages at Colorectal Cancer Diagnosis
Source: Radiol Res Pract. 2026 Aug 30;2026:6057943. doi: 10.1155/rrp/6057943 (PMC13527157; doi:10.1155/rrp/6057943)
Supplement: Supplementary file 1 [file RRP-2026-6057943-s002.docx]

**Supplementary Table 1 Coefficients from the regression models for each body composition parameter.**

|  | Age | | Sex | | Height | | P |
| --- | --- | --- | --- | --- | --- | --- | --- |
|  | β (SE) | 95% CI | β (SE) | 95% CI | β (SE) | 95% CI |  |
| SM volume  (cm^3^) | -9.84  (1.12) | -12.04, -7.65 | 509.65  (37.39) | 436.27, 583.02 | 24.65 (1.87) | 20.97, 28.33 | **<0.001** |
| SM Density (HU) | -0.41  (0.02) | -0.45, -0.36 | 6.14  (0.77) | 4.63, 7.65 | -0.18 (0.04) | -0.25, -0.10 | **<0.001** |
| SAT volume  (cm^3^) | -13.61 (4.74) | -22.91, -4.30 | -1507.12  (158.63) | -1818.42, -1195.83 | 54.57 (7.95) | 38.97, 70.17 | **<0.001** |
| SAT Density (HU) | 0.00  (0.03) | -0.05, 0.06 | 4.67  (0.96) | 2.79, 6.56 | -0.10 (0.05) | -0.19, -0.00 | **<0.001** |
| VAT volume  (cm^3^) | 26.79  (4.40) | 18.16, 35.42 | 892.77  (147.09) | 604.11, 1181.42 | 35.15  (7.37) | 20.69, 49.62 | **<0.001** |
| VAT Density (HU) | -0.02  (0.02) | -0.06, 0.03 | -1.55  (0.78) | -3.08, -0.01 | -0.05  (0.04) | -0.12, 0.03 | **0.001** |

**Supplementary Table 2 Residualized body composition measures by ACPS stage. Residuals were obtained from linear regression models adjusted for age, sex, and height. Values presented as mean ± SD of the standardized values for each stage. The p-value of each variable indicates the overall difference across all stages. Where this overall comparison was statistically significant, pairwise comparisons were performed, and only significant pairwise results are reported in the table.**

|  | Stage A  (n=234) | Stage B  (n=296) | Stage C  ((n=170) | Stage D  (n=270) | P value |
| --- | --- | --- | --- | --- | --- |
| SM volume  residual | 0.16 ± 0.95 | 0.05 ± 1.03 | -0.07 ± 0.91 | -0.19 ± 1.04 | **0.001**  A-D: <0.001  B-D: 0.03 |
| SM Density residual | 0.05 ± 1.02 | 0 ± 0.96 | -0.25 ± 1.06 | 0.11 ± 0.97 | **0.005**  A-C: 0.02  C-D: 0.003 |
| SAT volume  residual | 0.18 ± 0.88 | 0.02 ± 1 | 0.02 ± 0.97 | -0.24 ± 1.09 | **<0.001**  A-D: <0.001  B-D: 0.02  C-D: 0.05 |
| SAT Density residual | -0.28 ± 0.72 | -0.01 ± 0.94 | 0 ± 1.06 | 0.27 ± 1.19 | **<0.001**  A-B: 0.01  A-C: 0.04  A-D: <0.001  B-D: 0.01  C-D: 0.05 |
| VAT volume  residual | 0.26 ± 1.06 | -0.01 ± 0.96 | 0.03 ± 0.91 | -0.26 ± 0.98 | **<0.001**  A-B: 0.01  A-D: <0.001  B-D: 0.03  C-D: 0.02 |
| VAT Density residual | -0.26 ± 0.87 | -0.06 ± 1 | -0.01 ± 0.99 | 0.35 ± 1.04 | **<0.001**  A-D: <0.001  B-D: <0.001  C-D: 0.004 |
